# Effectiveness of a structured pharmacist-delivered intervention for patients post-acute coronary syndromes on all-cause hospitalizations and cardiac-related hospital readmissions: a prospective quasi-experimental study

**DOI:** 10.1007/s11096-023-01538-4

**Published:** 2023-02-16

**Authors:** Maguy Saffouh El Hajj, Rasha Kaddoura, Safae E. A. Abu Yousef, Bassant Orabi, Ahmed Awaisu, Sumaya AlYafei, Rula Shami, Ziyad R. Mahfoud

**Affiliations:** 1 Department of Clinical Pharmacy and Practice, College of Pharmacy, QU Health, Qatar University, 2713 Doha, Qatar; 2 Heart Hospital, Hamad Medical Corporation, 3050 Doha, Qatar; 3 College of Health Sciences, QU Health, Qatar University, 2713 Doha, Qatar; 4 World Innovation Summit for Health (WISH), Qatar Foundation, 5825 Doha, Qatar; 5 Weill Cornell Medicine-Qatar, P.O. Box 24144, Doha, Qatar

**Keywords:** Acute coronary syndrome, Hospitalization, Mortality, Patient discharge, Patient education, Pharmacist care, Qatar

## Abstract

**Background:**

Acute coronary syndrome (ACS) is a leading cause of mortality and morbidity in Qatar and globally.

**Aim:**

The primary objective of the study was to evaluate the effectiveness of a structured clinical pharmacist-delivered intervention on all-cause hospitalizations and cardiac-related readmissions in patients with ACS.

**Method:**

A prospective quasi-experimental study was conducted at Heart Hospital in Qatar. Discharged ACS patients were allocated to one of three study arms: (1) an intervention group (received a structured clinical pharmacist-delivered medication reconciliation and counselling at discharge, and two follow-up sessions at 4 weeks and 8 weeks post-discharge), (2) a usual care group (received the general usual care at discharge by clinical pharmacists) or, (3) a control group (discharged during weekends or after clinical pharmacists' working hours). Follow-up sessions for the intervention group were designed to re-educate and counsel patients about their medications, remind them about the importance of medication adherence, and answer any questions they may have. At the hospital, patients were allocated into one of the three groups based on intrinsic and natural allocation procedures. Recruitment of patients took place between March 2016 and December 2017. Data were analyzed based on intention-to-treat principles.

**Results:**

Three hundred seventy-three patients were enrolled in the study (intervention = 111, usual care = 120, control = 142). Unadjusted results showed that the odds of 6-month all-cause hospitalizations were significantly higher among the usual care (OR 2.034; 95% CI: 1.103–3.748, *p* = 0.023) and the control arms (OR 2.704; 95% CI: 1.456–5.022, *p* = 0.002) when compared to the intervention arm. Similarly, patients in the usual care arm (OR 2.304; 95% CI: 1.122–4.730, *p* = 0.023) and the control arm (OR 3.678; 95% CI: 1.802–7.506, *p* ≤ 0.001) had greater likelihood of cardiac-related readmissions at 6 months. After adjustment, these reductions were only significant for cardiac-related readmissions between control and intervention groups (OR 2.428; 95% CI: 1.116–5.282, *p* = 0.025).

**Conclusion:**

This study demonstrated the impact of a structured intervention by clinical pharmacists on cardiac-related readmissions at 6 months post-discharge in patients post-ACS. The impact of the intervention on all-cause hospitalization was not significant after adjustment for potential confounders. Large cost‐effective studies are required to determine the sustained impact of structured clinical pharmacist-provided interventions in ACS setting.

**Trial registration:**

Clinical Trials: NCT02648243 Registration date: January 7, 2016.

## Impact statements

- A structured comprehensive program provided by pharmacists to patients with ACS at hospital discharge and at follow-up can decrease cardiac readmissions at 6 months.
- Pharmacists can play an important role in the care of patients with ACS.
- The real impact of pharmacist interventions on other outcomes in ACS patients requires a well-designed randomized controlled trial.

## Introduction

Cardiovascular diseases (CVDs) are a leading cause of morbidity and mortality worldwide with an estimated 17.9 million CVD-related deaths in 2016, 85% of which were caused by stroke and acute coronary syndromes (ACS) [1]. Patients admitted with ACS are at a significant risk for recurrent cardiovascular and non-cardiovascular atherosclerotic events [2–4]. Therapeutic lifestyle modifications as well as secondary prevention therapies should be initiated in patients post-ACS [5–14]

Despite the availability of evidence-based clinical practice guidelines for in-hospital and post-discharge management of patients with ACS, there are gaps in the quality of care of these patients especially at discharge and during follow-up stages. For instance, if patients are not well educated about the importance of adherence to their secondary prevention medications, they are likely to discontinue these medications and to experience adverse health outcomes [15]. Evidence has shown that suboptimal use of and low adherence to these medications in ACS patients after hospital discharge are associated with significant negative consequences and economic burden to the healthcare system [16]. Furthermore, previous studies have documented the impact of pharmacists' care on patients post-ACS. These studies are summarized and synthesized in previous systematic reviews. However, most interventions in these studies were not well-structured as per prespecified protocols and did not utilize robust or powered study designs [17, 18]. Hence, the current study's rationale was to implement a structured comprehensive program involving a pharmacist for the care of patients post-ACS.

In Qatar, CVDs are the leading causes of mortality from non-communicable chronic diseases [19, 20]. According to the latest World Health Organization (WHO) data published in 2020, mortality due to coronary heart disease in Qatar reached 1,082 or 26.54% of total deaths [21].

Heart Hospital (HH) is the specialist governmental hospital for cardiology care in the country. It includes a state-of-the-art 20-bed coronary care unit,  a 12-bed cardiothoracic intensive care unit (ICU), a 24-bed surgical high-dependency unit (HDU), and a 60-bed ward [22]. In HH, around 3200 ACS cases are recorded each year [23–25]. Clinical pharmacists are an integral part of the healthcare system at HH. Their usual services include: attending clinical rounds with other members of the healthcare team, collaborating with physicians in the management of CVDs, optimizing the safety and effectiveness of patients’ pharmacotherapy, in addition to, performing medication reconciliation and patient education.

### Aim

This study was the first in Qatar to examine the effectiveness of a comprehensive structured clinical pharmacist-delivered intervention on cardiovascular-related outcomes in patients with ACS.

The primary objective was to evaluate the effectiveness of the structured clinical pharmacist-delivered intervention at hospital discharge and a tailored follow-up post-discharge on all-cause hospitalizations and cardiac-related hospital readmissions in ACS patients, 6 months post-discharge at HH. The secondary objectives of the study were to evaluate the effectiveness of the intervention, 6 months post-discharge on: all-cause emergency department (ED) visits, cardiac-related ED visits, all-cause mortality, cardiac-related mortality, and adherence to evidence-based secondary prevention therapy.

### Ethics approval

The study protocol and all related documents, including data collection forms, were reviewed and approved by the Institutional Review Board of the HMC’s Medical Research Center (approval number: Research Protocol 15319/15).

## Method

Details about the study methodology are available in a published protocol [26]. Below is a summary of the methods used in the study.

### Study design and setting

In the original study protocol we aimed to utilize an open-label, parallel-group, randomized, controlled trial (RCT) design. However, true participants’ randomization was not possible as it would have interfered with the care provided to patients in practice. Hence we conducted a prospective quasi-experimental study at HH in Doha, Qatar.

### Study eligibility criteria

Patients were included in the study if they were: (1) 18 years or older; (2) admitted to and discharged from any non-surgical cardiology ward or unit at HH with a diagnosis of ACS. Patients were excluded if they had any of following: severe visual impairment, severe hearing impairment, inability to communicate in English and/or Arabic, mental or psychiatric illnesses, delirium or severe dementia, cognitive impairment, incomprehensible speech, planned discharge to a location other than home, plan for coronary artery bypass graft (CABG) surgery during hospitalization, plan to leave Qatar in the next year, or a terminal illness with a high likelihood of death in the next year.

### Participants’ recruitment and allocation

Trained research assistants identified potential participants for the study by reviewing their hospital electronic files as per the study inclusion and exclusion criteria. Potentially eligible participants were approached, informed about the study, and invited to participate. After consenting, the patients’ sociodemographic information, medical and medication histories were collected. Patient recruitment was from March 2016 to December 2017.

True randomization of participants was not possible as this would have interfered with the flow of patients at the hospital; hence, intrinsic and natural allocation procedure for patients at the hospital was used. Upon admission to the emergency department at HH, patients diagnosed with ACS were typically assigned to different clinical teams (A, B, C and D) by a triage nurse, depending on the team’s capability. A clinical pharmacist was assigned to each team. Consenting patients who were assigned to the teams with intervention clinical pharmacists were automatically in the intervention arm, those who were assigned to the teams with non-intervention clinical pharmacists were considered in the usual care arm. In addition, patients who were discharged on weekends or on weekdays after the clinical pharmacists' working hours were automatically considered to be in the control arm.

### Intervention description

The study comprised three treatment groups:

1. Clinical pharmacist-delivered structured intervention *(intervention arm)*: In this arm, the clinical pharmacist offered a structured intervention at discharge, in addition to two 30-min coordinated follow-up sessions at 4 weeks and 8 weeks after hospital discharge.
  In brief, the intervention clinical pharmacist undertook the following activities:
  conducted medication reconciliation
  detected and resolved drug therapy problems (DTP)
  offered structured education and counselling to the patient
  communicated a follow-up medication monitoring plan to the patient after discharge
  provided the patient with a personalized medication timetable
  offered a pill box and instructed the patient how to fill it using the medication timetable as a guide
  provided the patient with patient information leaflets
  Follow-up sessions were held to reeducate and counsel the patients about the medications, remind them about the importance of adherence to medications, and answer any questions they may have.
2. *Clinical pharmacist-delivered usual care at discharge (usual care arm)*: In this arm, the clinical pharmacist offered the usual care to patients at discharge which included: medication reconciliation, identification and resolution of DTPs and discharge counseling.
3. *Regular discharge education by nurses and/or treating physicians (control arm)*: In this arm, patients obtained regular discharge education by nurses and/or treating physicians during hospital discharge. This arm included patients discharged during weekends and/or at times outside the clinical pharmacists' working hours.

### Study outcomes

The study research assistants, who were blinded to the study arms, documented the study outcomes at 6 months after discharge through assessing HH and HMC electronic medical records.

*Primary outcomes*:

1. all-cause hospitalizations
2. cardiac-related readmissions such as hospitalizations for new cardiac events, exacerbation of heart failure or arrhythmia.

*Secondary outcomes*:

1. all-cause ED visits including cardiac-related ED visits
2. all-cause mortality including cardiac-related mortality
3. cardiac-related mortality
4. adherence to evidence-based secondary prevention medications for CAD.

The patient adherence to secondary prevention medications was assessed using proportion of days covered (PDC) through the prescription refill records at the HH Outpatient Pharmacy covering the period from the patient discharge till the day of outcome assessment. The PDC considers the proportion of days in which the person has access to the medication, over the time frame and is calculated as: (sum of days covered in the time frame) ÷ (number of days in the time frame) × 100 [27]. Information about the patient ‘s medications including dosing regimens, prescribed daily dose refills, and therapeutic switches, were collected. Adjustment for prehospital supplies was not necessary. Patients who died were excluded (4 patients).

PDC was calculated for all secondary prevention medications (aspirin, β-blocker, statin, ACEI or ARB if applicable) that the patient was taking. A patient was considered adherent to drug therapy if the proportion of days covered (PDC) for each of the secondary prevention medications from discharge is larger than 75%. A PDC value of more than 75% was adapted based on published studies assessing adherence in patients with ACS [28].

### Sample size

The sample size was based on the primary analysis of comparing the main outcome among the three study arms. Based on the literature, assuming that the 6-month readmissions’ rate among the patients in the control arm is about 20% with 125 patients per arm [29], we considered that we will be able to detect an effect size of 0.025 between the three arms corresponding to an absolute decrease of 10% between the intervention arm and usual care arm and 9% between this latter arm and the control arm with a significance level of 5% and a power of 80% [26].

### Data analysis

Statistical analyses were conducted using the IBM SPSS (V.26, Armonk NY, USA). Intention-to-treat principle was used for the data analyses. The patients’ baseline characteristics between the arms were compared using one-way analysis of variance (ANOVA) test for numerical variables and χ^2^ test for categorical variables. Alternative non-parametric tests were used where applicable. Chi-square test was used to compare the three study arms in terms of the primary outcome measure (i.e., readmission rate). Pairwise comparisons using Bonferroni's adjustment for the significance level was used wherever applicable [30]. Furthermore, univariate binary logistic regression analysis was used to compute the unadjusted odds ratio (OR) and 95% CI of the OR between the three arms (with the intervention arm being the reference category). The number needed to treat (NNT) was computed when significance was achieved in the main outcome between any two pairs of arms. Secondary analysis involved multivariate binary logistic regression for the primary outcome, where adjustment was done for any imbalances in demographic and clinical variables or those deemed clinically important and a similar analysis for the secondary outcomes of all-cause mortality and adherence. In all statistical analyses, statistical significance was set at 0.05 except when multiple comparisons were done the Bonferroni’s adjustment was used.

## Results

**Fig. 1:**
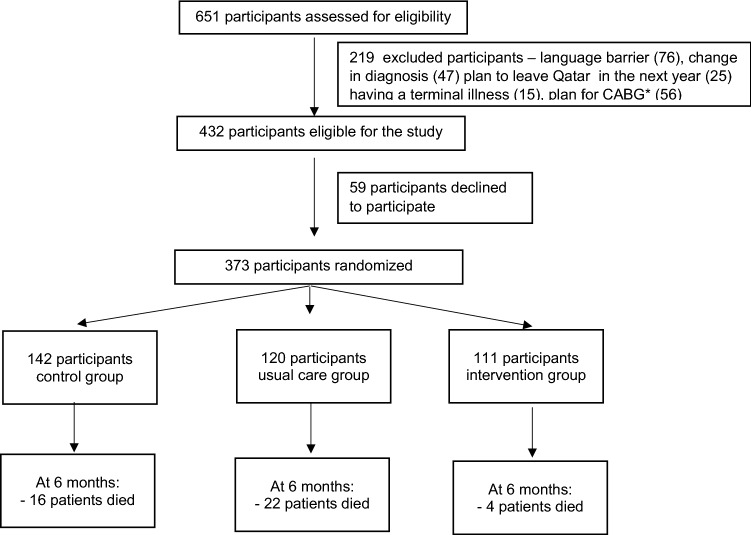
Participants screening and recruitment flow chart. *CABG: Coronary Artery Bypass Graft Surgery

Of 651 patients screened for eligibility in the study, 432 met the study inclusion criteria. A total of 373 patients consented to enroll in the study and were allocated into one of the three study arms (Fig. 1).

The baseline demographic and clinical characteristics of the study participants are presented in Table 1.

**Table 1:** Baseline demographic and clinical characteristics of patients post-acute coronary syndromes who received care in national Heart Hospital, Qatar (N = 373)

	Study arm			
	Control arm (n = 142)	Usual care arm (n = 120)	Intervention arm (n = 111)	*p* value
	n (%)	n (%)	n (%)	
Mean age (SD)	52.32 years (10.15)	54.78 years (11.54)	51.54 years (11.12)	0.06
*Gender*				
Male	125 (88%)	100 (83.3%)	106 (95.5%)	0.013*
Female	17 (12%)	20 (16.7%)	5 (4.5%)	
*Country of origin*				
Asian non-Arab countries	83 (58.5%)	58 (48.3%)	64 (57.7%)	0.318
African countries (North and South)	26 (18.3%)	18 (15%)	18 (16.2%)	
Other Arab countries	17 (12%)	20 (16.7%)	17 (15.3%)	
Qatar	14 (9.9%)	22 (18.3%)	9 (8.1%)	
Other	2 (1.4%)	2 (1.7%)	3 (2.7%)	
*Education level*				
Postgraduate university degree	5 (3.6%)	4 (3.4%)	5 (5.1%)	0.523
Undergraduate college degree	45 (32.6%)	42 (35.3%)	32 (32.3%)	
Secondary/high school	41 (29.7%)	28 (23.5%)	35 (35.4%)	
Less than secondary/high school	47 (34.1%)	45 (37.8%)	27 (27.3%)	
*Type of ACS*				
ST-elevation myocardial infarction	76 (53.5%)	46 (38.3%)	59 (53.2%)	0.016*
Non-ST-Elevation Myocardial Infarction	54 (38.0%)	52 (43.3%)	44 (39.6%)	
Unstable Angina	12 (8.5%)	22 (18.3%)	8 (7.2%)	
*Comorbidities*				
Diabetes mellitus	54 (38.0%)	65 (54.2%)	42 (37.8%)	0.013*
Heart failure	8 (5.6%)	8 (6.7%)	2 (1.8%)	0.181
Hyperlipidemia	21 (14.8%)	28 (23.3%)	12 (10.8%)	0.03*
Hypertension	56 (39.4%)	62 (51.7%)	40 (36.0%)	0.037*
Renal dysfunction	9 (6.3%)	11 (9.2%)	3 (2.7%)	0.124
Obesity	4 (2.8%)	7 (5.8%)	2 (1.8%)	0.267
Chronic obstructive pulmonary disease	4 (2.8%)	7 (5.8%)	6 (5.4%)	0.445
Cerebrovascular accident	3 (2.1%)	2 (1.7%)	1 (0.9%)	0.879
Coronary Artery Disease	31 (21.8%)	49 (40.8%)	16 (14.4%)	< 0.001*
*Cigarette smoking status*				
Current smoker	65 (45.8%)	40 (33.6%)	45 (41.3%)	0.129
Past smoker	20 (14.1%)	12 (10.1%)	11 (10.1%)	
Non-smoker	57 (40.1%)	67 (56.3%)	53 (48.6%)	
*Body Mass Index Category*				
Underweight (< 18.5)	1 (0.7%)	0 (0.0%)	0 (0.0%)	0.129
Normal weight (18.5–24.9)	38 (26.8%)	21 (17.5%)	27 (24.3%)	
Overweight (25.0–29.9)	53 (37.3%)	51 (42.5%)	54 (48.6%)	
Obesity (30 or more)	50 (35.2%)	48 (40.0%)	30 (27.0%)	
*Medical intervention for ACS*				
PCI (Percutaneous Coronary Intervention)	110 (77.5%)	78 (65.0%)	95 (85.6%)	0.004*
Medication Management Only	23 (16.2%)	33 (27.5%)	14 (12.6%)	
CABG (Coronary Artery Bypass Graft Surgery)	7 (4.9%)	3 (2.5%)	1 (0.9%)	
Other	2 (1.4%)	6 (5.0%)	1 (0.9%)	
*Medications on discharge*				
Beta blockers	128 (90.1%)	112 (93.3%)	105 (94.6%)	0.375
Aspirin alone	1 (0.7%)	3 (2.5%)	3 (2.7%)	0.393
Clopidogrel alone	1 (0.7%)	2 (1.7%)	5 (4.5%)	0.131
Ticagrelor alone	0 (0.0%)	1 (0.8%)	1 (0.9%)	0.527
Dual antiplatelets (Aspirin + clopidogrel or ticagrelor)	139 (97.9%)	113 (94.2%)	102 (91.9%)	0.089
Statin	137 (96.5%)	117 (97.5%)	105 (94.6%)	0.513
Angiotensin converting enzyme inhibitors/Angiotensin-receptor blockers	111 (78.2%)	92 (76.7%)	86 (77.5%)	0.959
Nitrates	120 (84.5%)	107 (89.2%)	101 (91.0%)	0.256
Proton Pump Inhibitors	58 (40.8%)	63 (52.5%)	46 (41.4%)	0.118
Diuretics	23 (16.2%)	35 (29.2%)	18 (16.2%)	0.015*
Calcium Channel Blockers	23 (16.2%)	22 (18.3%)	9 (8.1%)	0.067
*Presence of help at home*				
Yes	7 (5.0%)	8 (6.7%)	5 (4.7%)	0.754
No	134 (95.0%)	111 (93.3%)	102 (95.3%)	
Number of previous hospitalizations in the past 12 months Mean (SD)	0.32 (1.11)	0.57 (1.39)	0.25 (0.78)	0.078

*Statistically significant

Over half of the participants in the control arm (n = 76, 53.5%) and the intervention arm (n = 59, 53.2%) were admitted with ST-segment elevation myocardial infarction (STEMI), as compared with (n = 46, 38.4%) of the participants in the usual care arm (*p* = 0.016). Regarding the medical intervention for ACS, (n = 110, 77.5%), (n = 78, 65.0%), and (n = 95, 85.6%) of participants in the control, usual care, and intervention arms had PCI, respectively *p* = 0.004 (Table 1).

Unadjusted analyses of study outcomes are presented in Table 2. The rate of all-cause hospitalizations at 6 months was significantly lower in the intervention arm (n = 19, 17.1% of participants) as compared to usual care (n = 43, 35.8%) and control arms (n = 42, 29.6%) (*p* = 0.006). The NNT for all-cause hospitalizations was 6 for intervention versus usual care arm, and 9 for intervention versus control arm. Similarly, cardiac-related hospital readmissions at 6 months were also significantly lower in the intervention arm (n = 12,10.8%) then in the usual care (n = 37, 30.8%) and control arm (n = 31, 21.8%) (*p* = 0.001). NNT for cardiac-related hospital readmissions was 5 for intervention versus usual care arm, and 10 for intervention versus control arm. In addition, cardiac-related ED visits were significantly lower in the intervention arm (n = 4, 3.6%) as compared to usual care arm (n = 22,18.3%) and control arm (n = 16,11.3%) (*p* = 0.002). Cardiac-related mortality was significantly higher in the control arm (n = 4, 2.8%) than in the usual care and intervention arms (n = 0, 0%) (*p* = 0.038). In this study there were no patients who died of non-cardiac causes. Adherence to secondary preventive medications did not differ significantly among the three study arms (*p* = 0.156).

**Table 2:** Assessment of primary and secondary clinical outcomes among patients post-acute coronary syndromes who received care in national Heart Hospital, Qatar at 6 months (n = 373)

	Control arm (n = 142)	Usual care arm (n = 120)	Intervention arm (n = 111)	*p* value
	n (%)	n (%)	n (%)	
All cause-hospitalizations	42 (29.6%^**b**^)	43 (35.8%^**b**^)	19 (17.1%^**a**^)	0.006*
Cardiac-related hospital readmissions	31 (21.8%^**b**^)	37 (30.8%^**b**^)	12 (10.8%^**a**^)	0.001*
All cause-emergency department visits	39 (27.50%)	42 (35.00%)	33 (29.70%)	0.408
Cardiac-related emergency department visits	16 (11.3%^**b**^)	22 (18.3%^**b**^)	4 (3.6%^**a**^)	0.002*
Cardiac-related Mortality**	4 (2.8%^**b**^)	0 (0.0%^**a**^)	0 (0.0%^**a**^)	0.038*
Medication adherence	68 (50.0%)	72 (60.0%)	65 (60.7%)	0.156

^a,b^Arms with similar or common letters are not statistically significant from each other. Those with different letters are with *p* < 0.05

*Statistically significant

**In this study there were no patients who died of non-cardiac causes

The unadjusted and adjusted ORs for the primary outcome measures (all-cause hospitalizations and cardiac-related readmissions) are presented in Tables 3 and 4. At the bivariate level, the odds of 6-months all-cause hospitalizations were significantly higher among the usual (OR 2.034; 95% CI 1.103–3.748, *p* = 0.023) and control arms (OR 2.704; 95% CI 1.456–5.022, *p* = 0.002) as compared to the intervention arm. Similarly, patients in the usual care arm (OR 2.304; 95% CI 1.122–4.730,* p* = 0.023) and the control arm (OR 3.678; 95% CI 1.802–7.506, *p* ≤ 0.001) had greater likelihood of experiencing cardiac-related readmissions. When the data were adjusted for clinically important patients’ characteristics and imbalances at baseline (age, gender, PCI, CAD, diabetes, obesity, type of ACS, hypertension and hyperlipidemia), similar trends were observed. However, the only significant difference was observed between the control and the intervention arms for the 6 months cardiac-related readmissions outcome (OR 2.428:95% CI 1.116–5.282, *p* = 0.025) (Table 3). When the intervention arm was compared to the control and usual care arms combined, similar results were observed with increased odds of 6-month cardiac-related readmissions at the multivariate level (OR 2.140,; 95% CI 1.062–4.312 *p* = 0.033) (Table 4).

**Table 3:** Unadjusted and adjusted odds ratios of the primary outcomes for the three study groups

Arm	OR	95% C.I. for OR		*p* value	aOR^†^	95% C.I. for aOR		*p* value
		Lower	Upper			Lower	Upper	
*Cardiac-related readmissions at 6 months*								
Intervention arm	1				1			
Usual care arm	2.304	1.122	4.730	0.023*	1.939	0.913	4.117	0.085
Control arm	3.678	1.802	7.506	< 0.001*	2.428	1.116	5.282	0.025*

	OR	95% C.I. for AOR		*p* value	aOR^†^	95% C.I. for aOR		*p* value
		Lower	Upper			Lower	Upper	
*All-cause hospitalizations at 6 months*								
Intervention arm	1				1			
Usual care arm	2.034	1.103	3.748	0.023^*^	1.701	0.888	3.257	0.109
Control arm	2.704	1.456	5.022	0.002^*^	1.744	0.876	3.474	0.114

^†^Adjusted for age, gender, PCI, CAD, diabetes, obesity, type of ACS, hypertension, hyperlipidemia

*p value < 0.05

**Table 4:** Unadjusted and adjusted odds ratios for the primary study outcomes for the intervention group versus usual care plus control groups

Arm	OR	95% C.I. for OR		*p* value	aOR^†^	95% C.I. for aOR		*p* value
		Lower	Upper			Lower	Upper	
*Cardiac-related readmissions at 6 months*								
Intervention arm	1.000				1			
Usual care + control arm	2.892	1.495	5.593	0.002*	2.140	1.062	4.312	0.033*

Arm	OR	95% C.I. for OR		*p* value	aOR^†^	95% C.I. for aOR		*p* value
		Lower	Upper			Lower	Upper	
*All-cause hospitalizations at 6 months*								
Intervention arm	1.000				1			
Usual care + control arm	2.325	1.332	4.061	0.003*	1.719	0.941	3.138	0.078

^†^Adjusted for age, gender, PCI, CAD, diabetes, obesity, type of ACS, hyperlipidemia, hypertension

*p value < 0.05

## Discussion

To our knowledge, this was the first quasi-experimental study that evaluated the impact of a structured clinical pharmacist-delivered intervention at discharge and post-discharge for ACS patients in Qatar and probably in the Middle East. To put the results of this study in the context of previous similar studies synthesized in a systemic review by El Hajj et al. which included 17 studies (13 RCTs and four non-RCTs) pharmacist-delivered interventions significantly enhanced medication adherence in four out of 12 studies [17]. However, these did not translate into significant reductions in readmissions, hospitalizations, ED visits, and mortality in ACS patients [31–33].

It is worthwhile to note that in this study there were reductions in all-cause hospitalizations and cardiac-related readmissions in the intervention arm as compared to the control arm and usual care arm in the first adjusted model; however, these reductions were only significant for the outcome of cardiac-related readmissions between control care and intervention arms. There are several explanations for these findings. Despite the intrinsic allocation process, there were significant differences in some baseline characteristics between the participants in the three arms as the study was not a true randomized controlled clinical trial. Although these differences were controlled for through regression analysis, some unaccounted or unmeasured confounders whose effects were not evaluated may still have existed. In fact, the usual care arm tended to have more comorbidities and received more medical treatment than the intervention and control arms, which could have increased their chances of rehospitalization and cardiac events. In addition, it is plausible that some patients in the control arm could have received undocumented medication reconciliation and education by the nurses on duty and this might have affected the rate of readmissions and ED visits in this arm. In addition, failure to observe statistically significant results for some outcome measures might have been the results of not reaching the required sample size in each arm and thus decrease in the study power. For instance, the number of ACS patients in the intervention arm was relatively smaller than the estimated pre-study sample size of 125 patients per arm. This could have potentially decreased the power of the study and its ability to detect significant differences in outcomes. Another possible factor for these findings is related to the allocation of patients. The process of allocation of eligible patients in the study to the thee groups could have been more streamlined if true randomization was done. In comparison, other studies that assessed the effectiveness of pharmacist care in patients with ACS had similar or different explanations for their findings. For example, Bell et al. attributed the lack of statistically significant difference in their study outcomes to the high literacy level of patients who received the pharmacist care and to having non-clinical personnel contacting patients [34]. Kripalani et al. explained that their pharmacist intervention included highly educated patients which may have diluted the effect of the intervention [35]. Olson et al. indicated that their study was not powered enough to observe any significant differences between the groups in the study results [36]. On the other hand, the Medman study authors considered the following reasons for the study non-significant findings: patients receiving optimal care at baseline in addition to the inability to attain the study power and unavailability of information regarding practionners’ acceptability of pharmacists’ interventions [37].

Adherence to secondary prevention medications was assessed objectively using PDC which is the number of days a medication is filled divided by the days between fills [27]. Although the study found a significant positive impact of the intervention on hospital readmissions and ED visits, no significant differences in adherence were observed between the three arms. There are multiple reasons for this finding. One reason could be that participants might have had good health literacy at baseline, and consequently, this might have reduced the impact of the intervention. Patients with low health literacy are more likely to have unintentional non-adherence to medications than those with adequate health literacy, as a result, they are more likely to benefit from adherence interventions [38]. Stratification of patients according to health literacy or baseline medication adherence levels would have been beneficial [38]. Another possible reason for the lack of significant differences in adherence between the arms is that all patients attending government hospitals and clinics in Qatar pay a very small share of medication costs. Patients may get their prescription orders refilled but may not necessarily take the medications as instructed. Moreover, while using prescription refill records is a good measurement to assess adherence among patients, this technique does not guarantee that patients certainly took their medications. In our original plan, we aimed to also assess adherence by using the administration of Adherence to Refills and Medications Scale (ARMS) and through self-reported adherence; however, calling patients to assess their adherence through ARMS and self-report was limited by the unavailability of accurate phone numbers for several patients in the usual and control care arms.

### Strengths and limitations

The study had several strengths: the relatively long duration of follow-up, the presence of two comparison groups and the use of robust statistical adjustment models. Furthermore, the study employed a rigorous pharmacist-delivered intervention that was well-structured and intensive in nature. An additional strength was the use of different outcome measures to assess the impact of pharmacist intervention. The wide inclusion criteria and narrow exclusion criteria provided a study population that may be representative of Qatar’s population, which increased the generalizability of the study findings.

This study had some limitations. Assessing the study primary outcome was mainly based on HH and HMC electronic medical records. Hence, there was a possibility that the study participants could have visited private hospitals in case of an event or obtained their medications from outside pharmacies. We did not account for hospitalizations or emergency visits in private hospitals in Qatar, but this was unlikely to be a major limitation as HMC is Qatar’s main governmental institution and is the largest provider of care for Qatar citizens and residents.

Despite its limitations, this study reflected a real clinical practice and was among the few studies in the Middle East demonstrating the positive impact of pharmacist interventions on ACS patients. The results of the second regression model indicated that there was an absolute reduction in number of cardiac-related readmissions in the intervention arm as compared to the combined group of usual care and control arms. In addition, this study demonstrated the feasibility and the effectiveness of pharmacist-delivered interventions at and post discharge for ACS patients in Qatar and in the Middle East.

Furthermore, such interventions could have a favorable impact on the healthcare budgets since reducing hospital readmissions could result in reducing the use of healthcare resources, which could lead to cost savings. Several economic studies demonstrated that the various services provided by clinical pharmacists are cost-effective [39–42]. Future studies are needed to evaluate the economic impact of pharmacist-delivered interventions on Qatar’s healthcare system.

Collaboration with other healthcare providers is suggested and can further enhance the usefulness of such interventions. Studies have proven the effectiveness of interprofessional collaboration in reducing hospital readmissions [43–45], especially those involving pharmacists [46–48]. Therefore, we encourage the incorporation of other healthcare providers in the implementation of educational interventions led by pharmacists in order to benefit from their input.

## Conclusion

In summary, the study demonstrated the important role of pharmacists in the care of ACS patients. The study adjusted results showed that the intervention provided by clinical pharmacists significantly reduced cardiac-related readmissions at 6 months as compared to usual and control arms. Future larger, cost‐effective, multi‐center studies are required to assure the impact of the clinical pharmacist services on other outcomes in ACS patients.
